# Xenon improves long-term cognitive function, reduces neuronal loss and chronic neuroinflammation, and improves survival after traumatic brain injury in mice

**DOI:** 10.1016/j.bja.2019.02.032

**Published:** 2019-05-21

**Authors:** Rita Campos-Pires, Tobias Hirnet, Flavia Valeo, Bee Eng Ong, Konstantin Radyushkin, Jitka Aldhoun, Joanna Saville, Christopher J. Edge, Nicholas P. Franks, Serge C. Thal, Robert Dickinson

**Affiliations:** 1 Anaesthetics, Pain Medicine and Intensive Care Section, Department of Surgery and Cancer, UK; 2 Royal British Legion Centre for Blast Injury Studies, Department of Bioengineering, Imperial College London, UK; 3 Charing Cross Hospital Intensive Care Unit, Critical Care Directorate, Imperial College Healthcare NHS Trust, London, UK; 4 Department of Anaesthesiology, Medical Centre of Johannes Gutenberg University, Mainz, Germany; 5 Mouse Behavioural Outcome Unit, Focus Program Translational Neurosciences, Johannes Gutenberg University, Mainz, Germany; 6 Department of Life Sciences, Imperial College London, UK; 7 Department of Anaesthetics, Royal Berkshire Hospital NHS Foundation Trust, Reading, UK

**Keywords:** hippocampus, general anaesthesia, memory disorders, nerve degeneration, neuroinflammation, neuroprotection, traumatic brain injury

## Abstract

**Background:**

Xenon is a noble gas with neuroprotective properties that can improve short and long-term outcomes in young adult mice after controlled cortical impact. This follow-up study investigates the effects of xenon on very long-term outcomes and survival.

**Methods:**

C57BL/6N young adult male mice (*n*=72) received single controlled cortical impact or sham surgery and were treated with either xenon (75% Xe:25% O_2_) or control gas (75% N_2_:25% O_2_). Outcomes measured were: (i) 24 h lesion volume and neurological outcome score; (ii) contextual fear conditioning at 2 weeks and 20 months; (iii) corpus callosum white matter quantification; (iv) immunohistological assessment of neuroinflammation and neuronal loss; and (v) long-term survival.

**Results:**

Xenon treatment significantly reduced secondary injury (*P*<0.05), improved short-term vestibulomotor function (*P*<0.01), and prevented development of very late-onset traumatic brain injury (TBI)-related memory deficits. Xenon treatment reduced white matter loss in the contralateral corpus callosum and neuronal loss in the contralateral hippocampal CA1 and dentate gyrus areas at 20 months. Xenon's long-term neuroprotective effects were associated with a significant (*P*<0.05) reduction in neuroinflammation in multiple brain areas involved in associative memory, including reduction in reactive astrogliosis and microglial cell proliferation. Survival was improved significantly (*P*<0.05) in xenon-treated animals compared with untreated animals up to 12 months after injury.

**Conclusions:**

Xenon treatment after TBI results in very long-term improvements in clinically relevant outcomes and survival. Our findings support the idea that xenon treatment shortly after TBI may have long-term benefits in the treatment of brain trauma patients.

**Editor's key points**

• Xenon has neuroprotective effects, but its impact on long-term outcomes after traumatic brain injury has not been defined.
• In a mouse model of traumatic brain injury, short-term post-injury treatment with xenon reduced secondary injury and improved long-term neurological recovery and survival.
• This translational study suggests that xenon should be evaluated in human brain injury patients.

Traumatic brain injury (TBI) is a complex and heterogeneous disorder representing a significant global healthcare burden.^1^ In Europe, the annual incidence of TBI has been estimated at 326 per 100 000 population.^2^ Those patients who survive TBI frequently suffer from debilitating conditions that limit their ability to work or re-integrate to society, and have an increased risk of death. A recent long-term observational study on TBI patients aged 15–54 yr calculated a 7-fold increase in risk of death up to 13 yr after hospital admission.^3^, ^4^ In Europe, ∼7.7 million people are living with TBI-related disability.^5^ It is becoming clear that even a single TBI early in life is a risk factor for developing cognitive dysfunction, Alzheimer's disease (AD), and other neurodegenerative conditions later in life.^6^

Current clinical treatment for TBI patients is largely supportive, centred on non-specific endpoints such as management of tissue oxygenation, cerebral perfusion pressure and intracranial pressure.^7^, ^8^ At present, there are no clinically proven specific neuroprotective drugs for TBI.^9^, ^10^, ^11^ There is a need for neuroprotective treatments that minimise brain damage after TBI and improve long-term outcomes and survival.

Xenon is an anaesthetic noble gas that is neuroprotective in models of ischaemic brain injury. Xenon has recently been shown to be effective in reducing white matter damage in humans after ischaemic brain injury resulting from out-of-hospital cardiac arrest.^12^ Xenon is protective in *in vitro* models of brain trauma,^13^, ^14^, ^15^ but much less is known about its effects in brain trauma *in vivo.* We previously showed in an animal model that administration of xenon after TBI improves short-term (up to 4 days) and long-term outcomes (28 days) in mice.^16^ The current study is a longer-term follow-up that evaluates the effect of xenon administered to young adult male mice shortly after experimental brain trauma, investigating cognitive function, brain histology, and survival up to 20 months after TBI, which is approaching the end of a healthy animal's life span. We used the rodent controlled cortical impact (CCI) brain trauma model to test the following hypotheses: (1) xenon treatment after TBI improves very long-term cognitive and histological outcomes (at 20 months); and (2) xenon treatment after TBI improves survival at (i) 12 months and (ii) the end of the study (20 months).

## Methods

All experiments were approved by the Animal Ethics Committee of the Landestuntersuchungsamt Rheinland-Pfalz (protocol number: G12-1-010). We designed our study to comply with ARRIVE (Animal Research: Reporting of *In Vivo* Experiments) guidelines.^17^ Adult male C57BL/6N mice (*n*=77), aged 2.5 months, mean weight (standard error of the mean [sem]) 23.9 (0.1) g at the time of surgery or perfusion for naïve animals, were obtained from Charles River Laboratory (Sulzfeld, Germany). Animals were housed in filter-top cages in a pathogen-free facility in a 12:12 light–dark cycle (7 am–7 pm light) at 22°C with *ad libitum* access to food and water. Animals were closely monitored in the postoperative period for at least 4 h, and then early the following day. Long-term survival animals were checked at least once daily throughout the study.

### Experimental groups, randomisation, and blinding

Animals were randomly assigned to CCI primary injury (no treatment) or CCI followed by 75% Xe:25% O_2_ or CCI followed by 75% N_2_:25% O_2_ (control gas) or sham surgery followed by 75% N_2_:25% O_2_ (control gas) groups. The experimenter performing the CCI surgery was blinded to treatment. A separate experimenter, blinded to groups and treatment, performed behavioural tests. For cohort 1 (*n*=22), there were nine animals in each 24 h group (TBI control; TBI xenon) and four animals in the primary injury group. Animals were allowed to survive for 15 min (primary injury group) or 24 h. In cohort 2 (*n*=50), long-term (20 month) survival experiments had 20 animals each in the TBI xenon and TBI control groups, and 10 animals in the sham-surgery group. For cohort 3 (*n*=5), a naïve group of five animals aged 2.5 months was included for comparison of hemisphere volume and cerebral white matter with the long-term cohort.

### Traumatic brain injury

Animals were anaesthetised with 3.5 vol% sevoflurane in an air/oxygen mixture (40% O_2_:60% N_2_) supplied *via* a facemask in spontaneously breathing animals with buprenorphine analgesia (0.1 mg kg^−1^). Core body temperature was monitored and maintained at 37°C for the duration of the surgery by means of a rectal probe and feedback-controlled heating pad (Hugo Sachs, March-Hugstetten, Germany). Traumatic injury was performed using the CCI model, as described previously.^16^ Animals were fixed in a stereotactic frame (Kopf Instruments, Tujunga, CA, USA) and a 4×5 mm craniotomy window was created using a saline-cooled, high-speed drill along the coronal and lambdoid sutures and laterally as close as possible to the temporalis muscle insertion. The bone flap was lifted exposing the dura above the right parietal cortex, between the sagittal, lambdoid, and coronal sutures. The tip of a custom-built CCI device (L. Kopacz, Mainz, Germany) was positioned above the intact dura in the centre of the craniotomy window (1 mm from sagittal suture and 1 mm from lambdoid suture). The angle of the impactor, typically 25° from the sagittal plane, was adjusted such that the tip was perpendicular to the dural surface. The impactor tip was flat, with a diameter of 3 mm, impact velocity of 8 m s^−1^, impact duration of 150 ms, and penetration depth of 1.0 mm. Our CCI impact parameters and the functional and histological outcomes are similar to those classified as a moderate–severe injury.^18^ After CCI surgery, the craniotomy was closed with the bone flap, sealed with tissue glue (Histoacryl; Braun-Melsungen, Melsungen, Germany), and the skin sutured. Sham-surgery animals underwent identical anaesthesia, temperature control, placement in stereotaxic frame, surgical skin incision to reveal the surface of the skull, which was drilled superficially, but no craniotomy was performed. The duration of the sham surgery and anaesthesia was identical to that of the CCI animals. In order to avoid any confounding effects from the anaesthesia and analgesia we were careful to ensure that the sham group received exactly the same drugs. The choice of anaesthetic and analgesic drugs in animal TBI studies may also have an impact on how secondary injury develops. For our study we chose to combine sevoflurane and buprenorphine. Sevoflurane has been shown to have minimal effects on secondary injury development in TBI models.^19^ Buprenorphine is a highly effective and safe analgesic in rodents.^20^, ^21^ Despite some controversy surrounding opioid effects on TBI outcomes in rodents,^22^, ^23^ evidence is limited. Interestingly, buprenorphine has been shown to have no impact on injury development in rodent models of brain ischaemia.^24^, ^25^ Overall, the benefits of appropriate intraoperative and postoperative analgesia are well described, and mandatory for ethical and animal welfare. Animals were returned to their individual home cages in a heated incubator (33°C, 35% humidity; IC8000; Draeger, Lübeck, Germany) and allowed to recover for 15 min before treatment, breathing room air.

### Xenon or control gas administration

Gas treatments were administered to spontaneously breathing animals in a series of custom-made xenon exposure chambers linked in a closed circuit, for a total duration of 3 h, starting 15 min after CCI injury. Gas concentrations inside the circuit were monitored continuously *via* a xenon meter (model 439 EX; Nyquist Ltd, Congleton, UK) and an oxygen meter (Oxydig; Draeger). Carbon dioxide was removed from the system using soda lime pellets. Additional volumes of gases were added as necessary to maintain concentrations of 21–25% for oxygen and 70–75% for xenon throughout the 3 h administration period. Gases were circulated at 700 ml min^−1^ by a small animal ventilator (Inspira ASV; Harvard Apparatus, Holliston, MA, USA). Xenon (BOC HiQ 74.96% xenon: 25.04% oxygen) was obtained from BOC Ltd (Guildford, UK). The exposure chambers were housed inside a heated incubator. After the 3 h treatment with xenon or control gas, animals were returned to the home cage where they breathed room air.

### Quantification of functional outcome

#### Neuroscore

Functional outcome before and after CCI injury was determined using a 15-point neurological outcome score evaluating locomotor ability, vestibulomotor function, and general behaviour.^16^, ^26^ The neuroscore was performed in real time before CCI surgery and repeated 24 h after injury by an experimenter blinded to the surgical and treatment groups. Because the sham-surgery animals had the same skin incision and sutures as the CCI animals, they were not visually distinguishable.

#### Contextual fear conditioning

Two weeks and 20 months after the injury, cognitive function was assessed in the same cohort of animals by a contextual fear conditioning test implemented using a multi-conditioning system (TSE Systems GmbH, Bad Homburg, Germany). The test measures hippocampus-dependent contextual place memory. For the 2 week and 20 month tests, entirely different contexts (chambers) were used in order to test memory of novel environments. These tests were carried out by another blinded observer. For context conditioning training, an animal was placed inside a conditioning context Plexiglas chamber (36×20×20 cm) with a removable shock grid made of stainless steel rods (4 mm in diameter, spaced 6 mm apart). After 2 min, animals received a first electrical shock (0.4 mA, 2 s) and 15 s later a second shock with the same characteristics. Animal behaviour was recorded by a video camera to monitor freezing behaviour, defined as the lack of movement (excluding respiratory movements). The analysis of the freezing behaviour was performed using computerised video software (EthovisionXT software; Noldus Information Technology, Wageningen, The Netherlands). The contextual memory test was performed 24 h after context conditioning training. Mice were monitored for freezing for 2 min in the same context chamber that was used for training. The cumulative duration (s) of freezing behaviour during the 2 min of testing was used.

#### Survival analysis

The long-term survival cohort was kept for 20 months after CCI surgery to assess survival. Animals were monitored on a daily basis. Deaths that occurred up to the end of the observation protocol were spontaneous, and no animals had to be euthanised.

### Histological processing

At 15 min and 24 h animals were anaesthetised with sevoflurane and euthanised by cervical dislocation. Brains were carefully removed, frozen on powdered dry ice, and stored at –80°C. At 20 months the surviving cohort of animals were terminally anaesthetised with pentobarbital and transcardially perfused with 20 ml of cold phosphate-buffered saline (PBS; Biochrom GmbH, Berlin, Germany) followed by 30 ml of cold 4% paraformaldehyde (DAC paraformaldehyde; Merck KGaA, Darmstadt, Germany). Brains were carefully removed from the skull and post-fixed in 4% paraformaldehyde (in PBS) overnight at 4°C, then transferred to 30% sucrose in PBS until the brains sank, before being frozen as described above. Naïve animals were terminally anaesthetised and perfused in the same manner. Frozen brains were embedded in Optimal Cutting Temperature mounting medium (Cell Path Ltd, Newton, Powys, UK) and cut in the coronal plane with a cryostat tissue slicer (Microm HM 560 [15 min, 24 h] or CryoStar NX70 [20 month, naïve]; Thermo Scientific, Walldorf, Germany). To quantify lesion volume, for each brain a total of 16–18 sections (10 μm thick) spanning the entire lesion were collected on Superfrost^®^ Plus microscope slides (Thermo Fisher Scientific, Braunschweig, Germany) every 500 μm, starting at Bregma +3.14 mm. For immunohistochemistry on the long-term brains, 20 μm-thick slices were also obtained at a brain point 4.5 mm from the anterior pole of each brain (because of variable atrophy in the aged brains, these slices ranged from Bregma –1.25 and –2.15 mm when mapped to the Allen mouse brain atlas).^27^, ^28^

#### Quantification of contusion volume

Slices (10 μm) were stained with cresyl violet (Merck Millipore, Darmstadt, Germany) as described.^16^ Slices were imaged with a digital camera (Scopetek DCM510; Scopetek Opto-Eletric Co. [Hangzhou, China] or Zeiss Axiocam 105 colour; Zeiss [Jena, Germany]) attached to a stereomicroscope (Wild model M8, Heerbrugg, Switzerland or Zeiss Stemi 305, Zeiss). The contusion was evident from a clear difference in the intensity of the cresyl staining. The area of the contusion was measured using image analysis software (Scopephoto 3.1, Scopetek Opto-Eletric Co. or Zen, Zeiss) by an investigator blinded to the experimental groups. Contusion volume was calculated by multiplying contusion areas, *A*, by the distance between brain sections, *d* (500 μm), according to the following formula:

$$\frac{d}{2}*\left(A_{1\;}+A_{n}\right)+d*\left(A_{2\;}+A_{3}+\ldots +A_{n-1}\right)$$

Secondary injury volume at 24 h was calculated by subtracting the mean primary injury contusion volume at 15 min from the total contusion volume measured at 24 h.

#### Quantification of area of myelinated fibres in the corpus callosum

Slices (10 μm) were stained overnight with 1% w/v Luxol Fast Blue in 95% isopropanol:5% acetic acid (10%) at 60°C in a humidified water bath. Slices were washed briefly in 95% isopropanol followed by distilled water. Slices were differentiated in 0.5% w/v lithium carbonate for 30 s, followed by 70% isopropanol for 30 s followed by distilled water, then counterstained with cresyl violet. Slices (Bregma –1.94 mm) were imaged with a digital camera (Zeiss Axiocam 105 colour; Zeiss) attached to a stereomicroscope (Zeiss Stemi 305, Zeiss). The area of the contralateral corpus callosum was measured by a blinded investigator from the midline to a point directly above the distal end of the dentate gyrus (DG) granule cell layer^29^ using the area measurement function in ImageJ (FIJI).^30^

#### Immunofluorescence staining and analysis

Twenty micrometre-thick slices from the perfused brains of the surviving cohort of animals were used for immunofluorescence staining for glial fibrillary associated protein (GFAP, reactive astrocytes), ionised calcium-binding adapter molecule 1 (Iba1, microglia), neuronal nuclei (NeuN, neurones), and 4′,6-diamidino-2-phenylindole (DAPI) (nuclei). GFAP: slices were washed in PBS for 3 min and blocked for 1 h with 5% normal goat serum, 0.5% bovine serum albumin (diluted in PBS–1% Tween). Sections were incubated overnight at 4°C with rat anti-mouse GFAP (1:500 in blocking solution, 13-0300 Invitrogen/Life Technologies, Carlsbad, CA, USA). The following day, sections were washed with PBS–1% Tween and incubated for 2 h at room temperature with Alexa Fluor^®^ 568 goat anti-rat (A-11077 Invitrogen/Life Technologies). Sections were washed in PBS–1% Tween, incubated with DAPI (1:10000 in PBS–1% Tween) for 5 min and mounted using Shandon Immunomount^®^ (Thermofisher, Schwerte, Germany). Iba1 and NeuN: after antigen retrieval in 0.015 M citric acid, pH 6.0, at 75°C for 20 min, slices were allowed to cool, and washed in PBS for 1 min then blocked for 1 h with 5% normal goat serum, 1% bovine serum albumin (diluted in PBS–0.1% TritonX). Sections were incubated overnight at 4°C with rabbit anti-Iba1 (1:500 in blocking solution, 019-19741; Wako, Neuss, Germany) and mouse anti-NeuN (1:500 in blocking solution, MAB377 Millipore, Darmstadt, Germany). The following day, sections were washed with PBS and incubated for 2 h at room temperature with Alexa Fluor^®^ 568 goat anti-rabbit (1:500, A11011 Invitrogen/Life Technologies) and Alexa Fluor^®^ 488 goat anti-mouse (1:500, A11001 Invitrogen/Life Technologies). Sections were washed in PBS–1% Tween, incubated with DAPI (1:10000 in PBS–0.1% TritonX) for 5 min, then washed in PBS and mounted using Shandon Immunomount^®^ (Thermofisher). The stained sections were imaged using an Axio Observer Z1 widefield microscope (Carl Zeiss AG, Oberkochen, Germany) with a 20× (GFAP) or 10× (NeuN; Iba1) objective (20× Zeiss Plan-Apochromat NA 0.80, WD 0.55 mm; 10× Zeiss EC Plan-Neofluar NA 0.30, WD 5.20 mm). Whole sections were imaged using the multi-photon acquisition function of Zeiss Zen software (excitation and emission wavelengths: DAPI 335–383, 420–470 nm; Iba1 and GFAP 538–562, 570–640 nm; NeuN 450–490, 500–550 nm). Images were analysed by blinded investigators using ImageJ (FIJI). The GFAP positive area was quantified as percentage area (%-area) in the corpus callosum and in defined regions of interests (ROIs) (Fig. 1a) in the hypothalamus, retrosplenial cortex, amygdala, and hippocampal CA1, CA2, CA3, and DG regions, after rolling ball background subtraction and thresholding (equally for all images) to best match the raw image. For quantification of GFAP positive scar bordering the lesion cavity, images were thresholded (equally for all images) to best match the raw image and the area of interest delineated with a polygon selection in the retrosplenial and the somatosensory cortex. The GFAP-positive total stained area (μm^2^) and the length (μm) of the ROI were used to calculate an average scar thickness (μm) at the lesion cavity using ImageJ (FIJI). GFAP is upregulated when astrocytes become reactive, and is a reliable and sensitive marker of most reactive astrocytes.^31^ Because we were interested in quantifying reactive astrogliosis we chose to measure the percentage area of GFAP positive immunoreactivity, rather than count the absolute number of astrocytes. The number of Iba1 positive cells was manually counted in the same defined ROIs as above (Fig. 1a). NeuN positive cells were manually counted in the same ROIs as above for hypothalamus, retrosplenial cortex, and amygdala. For quantification of NeuN positive cells in the hippocampus, we used rectangular ROIs, parallel to and centred on the pyramidal cell layers—CA1 300×50 μm, CA2 200×50 μm, CA3 150×300 μm, and DG 2× 300×80 μm boxes—one in each arm. In all of the long-term histology, TBI control and TBI xenon groups were compared with the sham group that was the same age and had been treated identically to the TBI groups but without impact, in order to ensure that any effects are independent of age-related changes in particular cell types or immunoreactivity.

**Fig. 1:**
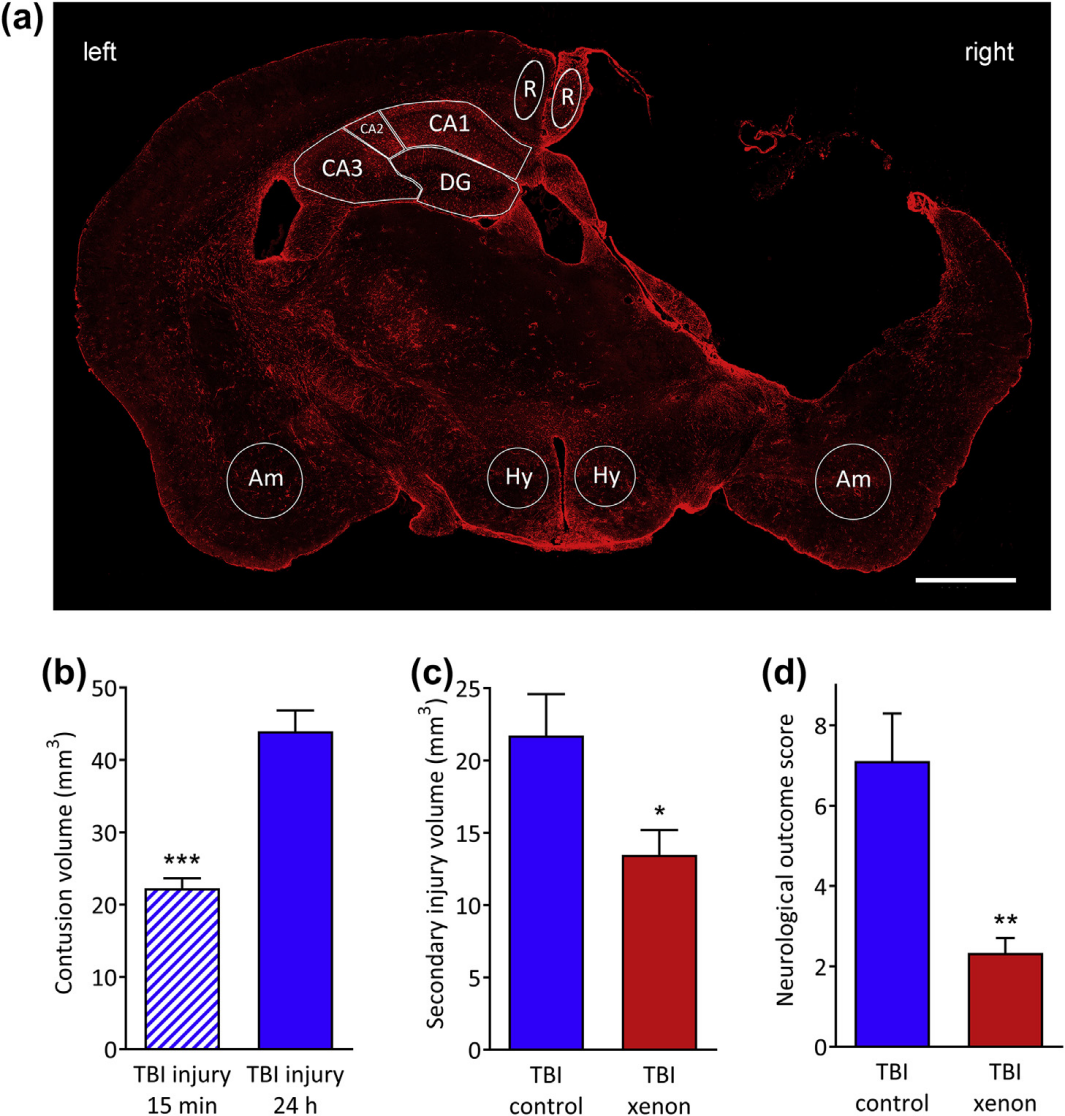
(a) A typical GFAP stained brain slice at Bregma –1.75 mm from a TBI control animal 20 months after injury. GFAP positive area was measured in the retrosplenial cortex (R), hypothalamus (Hy), amygdala (Am) and the hippocampus (CA1, CA2, CA3, and DG regions). The lesion on the right side of the cortex is clearly visible on the right of the image. For the retrosplenial cortex the dimensions of the region of interest (ROI) was an ellipse of 240 μm (width) × 605 μm (height); for the hypothalamus the ROI was a circle of diameter 600 μm; for the amygdala the ROI was a circle of diameter 750 μm; the ROIs for the hippocampus were drawn manually for each slice (using the Allen mouse brain atlas^28^ as a reference). Scale bar, 1000 μm. (b) Acute phase injury development. The controlled cortical impact model produces a primary lesion, clearly seen 15 min after trauma (hatched blue bar), with injury developing significantly 24 h after impact (control gas treatment); (*n*=4, TBI 15 min; *n*=9, TBI 24 h). (c) Xenon (red bar) significantly reduces secondary injury volume at 24 h compared with control gas (blue bar). Secondary injury is calculated by subtracting primary injury (15 min) from total contusion volume at 24 h (*n*=9, TBI control; *n*=9, TBI xenon). (d) Xenon improves short-term neurological outcome 24 h after injury (*n*=9, TBI control; *n*=9, TBI xenon). Control gas-treated animals (blue solid bars) received 75% nitrogen:25% oxygen. Xenon-treated animals (red bars) received 75% xenon:25% oxygen. Treatment was started 15 min after the impact and was administered for 3 h. Bars represent mean values and error bars are standard errors. *P<0.05; **P<0.01; ***P<0.001, compared with TBI control at 24 h. GFAP, glial fibrillary associated protein; DG, dentate gyrus; TBI, traumatic brain injury.

### Statistics

Data were assessed for normality using the Shapiro–Wilk test. We assessed significance of differences in contusion volume, secondary injury volume, and neurological outcome score between TBI control and TBI xenon groups using a two-tailed Student's *t*-test. For the fear conditioning test and hemisphere volume data, we compared sham, TBI control, and TBI xenon groups using two-way analysis of variance (anova) or one-way anova, respectively, with Bonferroni's *post hoc* test. Data on corpus callosum area, GFAP positive area, and Iba1 in the corpus callosum were analysed using one-way anova with Bonferroni's *post hoc* test. In order to test the hypothesis that xenon treatment reduces astrocytic scarring, the GFAP positive scar data were analysed using a one-tailed Student's *t*-test. Normality tests, anova, and Student's *t*-tests were implemented using Graphpad Prism Version 7 software (GraphPad Software Inc., La Jolla, CA, USA). Some of the NeuN, Iba1, and GFAP positive distributions in the ROIs in the hypothalamus, retrosplenial cortex, amygdala, and hippocampus were significantly different from a normal distribution, and could not be transformed into a normal distribution. Therefore, the long-term histology of these areas in the TBI control, TBI xenon and sham groups were compared using a Kruskal–Wallis (KW) test with Benjamini–Yekutieli correction for multiple comparisons implemented using the statistical program Stata (Version 15; StataCorp, College Station, TX, USA). As the null statistics for the KW test are known not to follow a χ^2^ distribution for small numbers especially in the region of the 0.95 and 0.99 quantile, results from the KW test were compared with the exact results for a KW test using a program written in Mathematica (Mathematica 11.3.0.0; Wolfram Research Inc., Champaign, IL, USA).^32^ For long-term survival data, cumulative event curves were constructed for the survival of animals with the use of the Kaplan–Meier method using GraphPad Prism. A one-tailed Gehhan–Breslow–Wilcoxon test, with the Bonferroni–Holm correction, was used to assess differences between survival curves for TBI control and TBI xenon groups at 12 and 20 months after injury. The hazard ratio was calculated using the method of Mantel–Haenszel. A *P*-value of 0.05 or less was taken to indicate a significant difference. Values are quoted as mean (sem) for normally distributed data or median (inter-quartile range) if data are not normally distributed. Sample sizes (*n*) are indicated in the figure legends.

## Results

### Xenon treatment reduces secondary injury and improves outcome acutely after TBI

The injury parameters we used create a primary injury such that secondary injury develops significantly (*P*<0.001), increasing by 98% between 15 min and 24 h after trauma (Fig. 1b) in the TBI control group. Xenon treatment, starting 15 min after injury for 3 h, resulted in a significant reduction in secondary injury by 38% (5%) (*P*<0.05) at 24 h after injury (Fig. 1c). The reduction in secondary injury volume at 24 h translated into significant improvement in neurological outcome, with a 67% (11%) reduction (*P*<0.01) in neurological impairment score (Fig. 1d). Neurological outcome score was zero in all groups before CCI or sham surgery, and in the sham-surgery group 24 h later (data not shown).

### TBI in young adult mice results in late-onset cognitive impairment that is prevented by xenon treatment

We investigated development of the cognitive phenotype at different time points after TBI in the same experimental cohort. We therefore investigated cognitive performance at 2 weeks and 20 months after injury, using a different contextual fear conditioning paradigm at each time point to investigate associative memory. At 2 weeks after injury, duration of immobilisation or ‘freezing’ in the pre-trial before fear conditioning was low for all groups (∼6 s), and there was no significant difference between groups (Fig. 2a). At 24 h after the fear conditioning in context trial, all groups exhibited increased ‘freezing’ behaviour (∼12 s) compared with pre-trial (*P*<0.001), indicating that they had remembered the context. However, at 2 weeks after injury there was no difference in the duration of freezing between groups (Fig. 2a), indicating all groups recalled the context to the same extent. The lack of difference in freezing time between sham and either of the TBI groups indicates that there was no early memory impairment. At the 20 month time point freezing time in the pre-trial was also low (<6 s) for all groups with no significant differences between groups. However, at the 20 month time point, in the context trial there was a significant reduction (*P*<0.05) in freezing duration in the TBI control group [29 (4) s] compared with the uninjured sham group [47 (3) s], indicating a late-onset memory impairment in the TBI control group (Fig. 2b). Interestingly, at the 20 month time point the xenon-treated TBI group showed no memory impairment as indicated by the fact that freezing duration [41 (5) s] was not significantly different to that of the sham group (Fig. 2b). This suggests that early xenon treatment after TBI prevents late-onset hippocampus-dependent memory impairment.

**Fig. 2:**
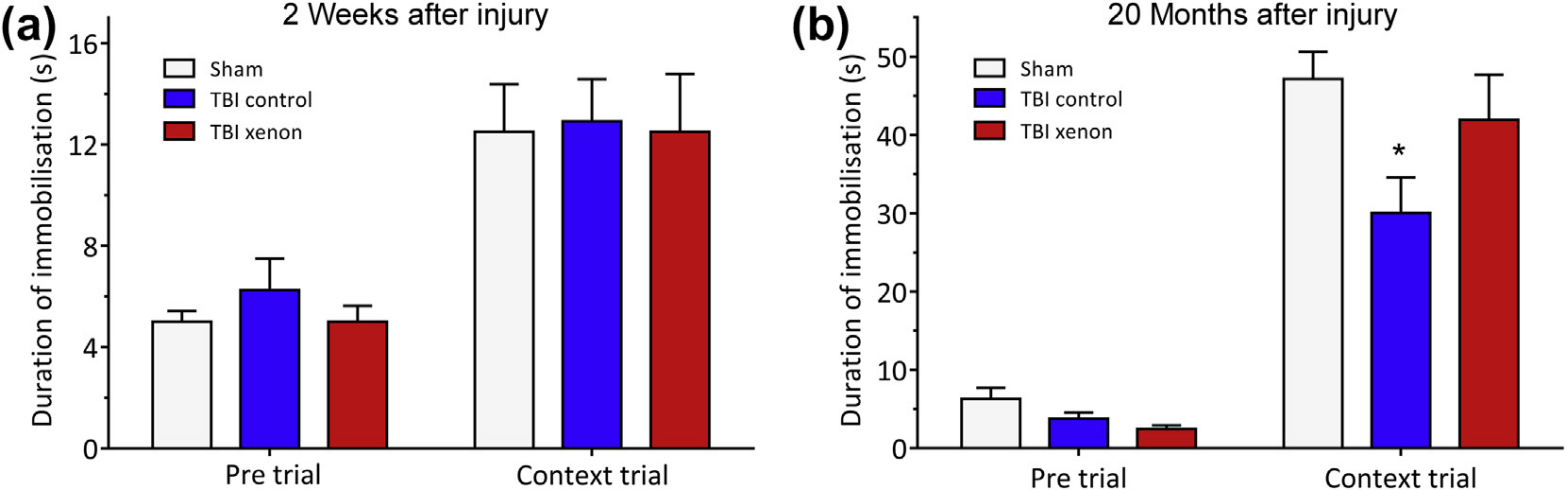
Xenon treatment shortly after TBI prevents the development of late-onset TBI-related memory deficits in the same cohort. (a) At 2 weeks after injury, in a contextual fear conditioning test, no differences between groups in freezing behaviour were observed in the context-trial period indicating there were no memory impairments in either TBI group. (b) At 20 months after injury there was a significant memory impairment in the untreated TBI control group compared with the sham group. Xenon treatment prevented the onset of this impairment. No differences between groups were observed in the pre-trial period. During contextual trial, there was a significant reduction in freezing time in the TBI control group, indicating a memory deficit, compared with the uninjured sham group. Freezing time in TBI xenon group was not significantly different to the sham group. TBI control (blue bars) and sham operated animals (white bars) received 75% nitrogen:25% oxygen; TBI xenon animals (red bars) received 75% xenon:25% oxygen. Treatment was started 15 min after the impact or sham procedure and was administered for 3 h. Bars represent mean values and error bars are standard errors (2 weeks: *n*=10, sham; *n*=20, TBI control; *n*=20, TBI xenon; 20 months: *n*=7, sham; *n*=9, TBI control; *n*=13, TBI xenon). *P<0.05 compared with sham. TBI, traumatic brain injury.

### Brain volume and lesion size 20 months after injury

Compared with young adult naïve brains the brain hemisphere volumes of all groups were significantly (*P*<0.0001) smaller (Supplementary Fig. S1). The left and right hemisphere volumes in the sham group were respectively 28.1% (0.5%) and 30.5% (0.2%) smaller compared with the young naïve group. This is most likely caused by brain atrophy as a result of ageing, but we cannot exclude the possibility that anaesthesia and sham surgery are contributory factors. In the TBI control and TBI xenon groups, the contralateral (left) hemispheres were 30.3% (0.4%) and 27.8% (0.2%) smaller than the young naïve group. Traumatic lesions were present at 20 months in both TBI groups, but there was no difference in volume between the TBI control group [17.7 (1.9) mm^3^, *n*=8] and the xenon-treated TBI group [17.6 (1.6) mm^3^, *n*=11].

### Xenon treatment reduces long-term white matter degeneration after TBI

**Fig. 3:**
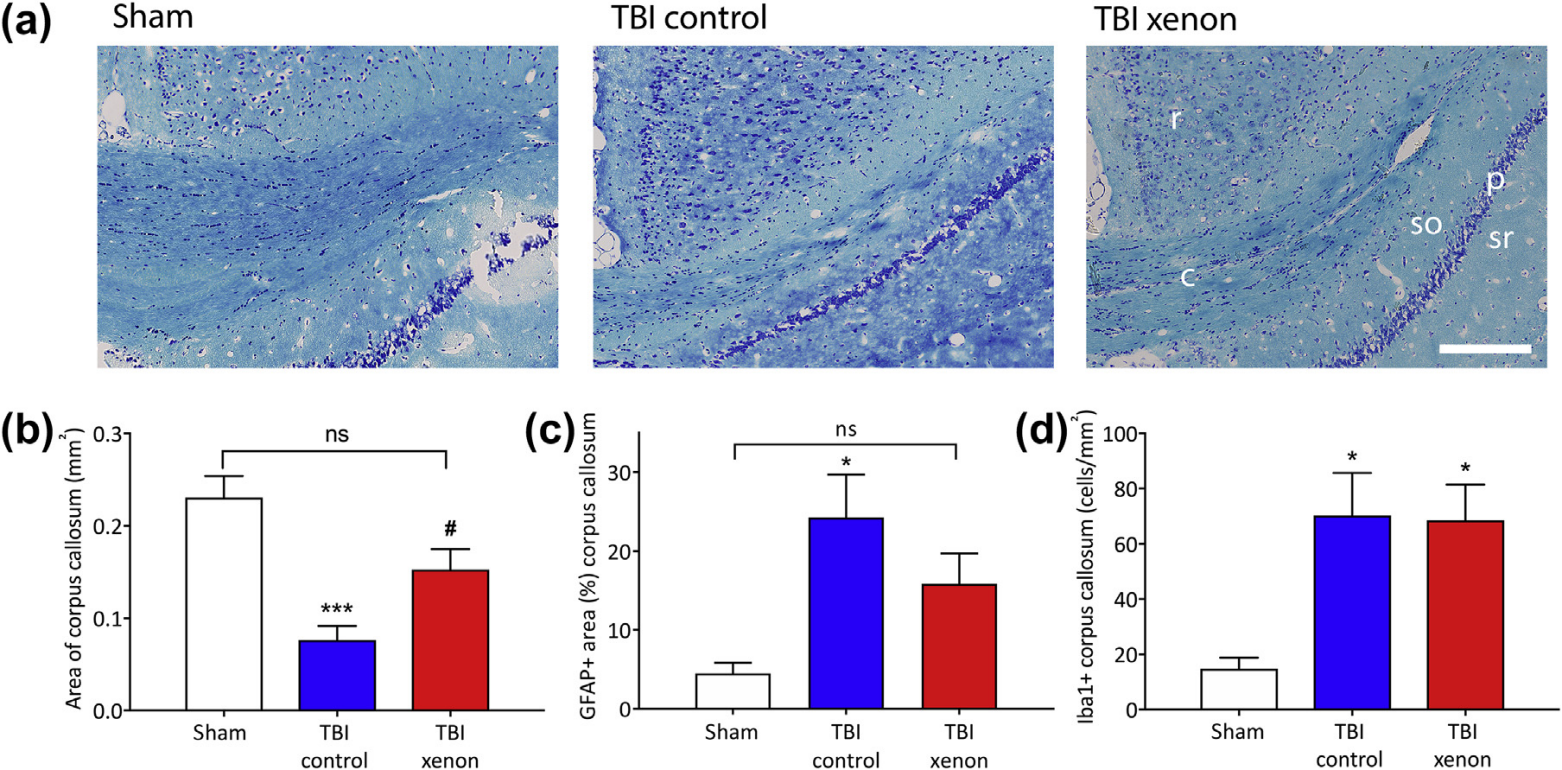
Xenon treatment after TBI reduces long-term white matter degeneration in the contralateral corpus callosum 20 months after injury. (a) Typical Luxol Fast Blue stained slices showing myelinated fibres (dark blue) in the contralateral corpus callosum. Scale bar, 200 μm. r, retrosplenial cortex; c, corpus callosum myelinated fibres; so, stratum oriens (CA1); p, pyramidal layer (CA1); sr, stratum radiatum (CA1). (b) There was a significant reduction in the area of myelinated fibres in contralateral corpus callosum in the TBI control group compared with the sham group. Xenon treatment after TBI reduced the white matter loss in corpus callosum (corpus callosum area: *n*=7, sham; *n*=8, TBI control; *n*=11, TBI xenon). (c) There was a significant increase in GFAP positive staining in the contralateral corpus callosum in the TBI control group compared with the sham group, consistent with astrogliosis. The TBI xenon group was not significantly different to the sham group (GFAP area: *n*=7, sham; *n*=7, TBI control; *n*=11, TBI xenon). (d) The number of Iba1 positive cells was significantly increased in both TBI control and TBI xenon groups compared with the sham group (Iba1: *n*=6, sham; *n*=7, TBI control; *n*=10, TBI xenon). TBI control animals (blue bars) and sham animals (white bars) received 75% nitrogen:25% oxygen. TBI xenon animals (red bars) received 75% xenon:25% oxygen. Treatment was started 15 min after the impact or sham procedure and was administered for 3 h. The bars indicate mean values and the error bars are sem. *P<0.05, ***P<0.001 compared with sham; #*P*<0.05 compared with TBI control. GFAP, glial fibrillary associated protein; TBI, traumatic brain injury; sem, standard error of the mean.

In order to determine the effect of injury and xenon treatment on neuronal white matter 20 months after injury, we measured the area of myelinated fibre tracts stained with Luxol Fast Blue in the contralateral corpus callosum (Fig. 3a and b). The area of the corpus callosum in the sham group at 20 months was 0.23 (0.02) mm^2^, which was not significantly different to that of the naïve group, aged 2.5 months, 0.20 (0.04) mm^2^ (data not shown), indicating that in the absence of trauma there was no age-related neurodegeneration. However, in the TBI control group we observed a significant (*P*<0.001) reduction of 67% (13%) in white matter compared with the uninjured sham group. In contrast, the area of the contralateral corpus callosum myelinated fibre tracts in the TBI xenon group was significantly (*P*<0.05) larger, by 100% (14%), than the TBI control group and was not significantly different to the uninjured sham group. The reduction in white matter in the TBI control group was accompanied by a significant (*P*<0.05) increase in GFAP positive astrocytes in the corpus callosum compared with the sham group, whereas in the xenon-treated TBI group GFAP positive staining was not significantly different to the sham group (Fig. 3c). There was a significant (*P*<0.05) increase in Iba1 positive microglia in the corpus callosum in both the TBI control and TBI xenon groups compared with the sham group (Fig. 3d).

### Xenon treatment reduces chronic perilesional astrocytic scarring after TBI

**Fig. 4:**
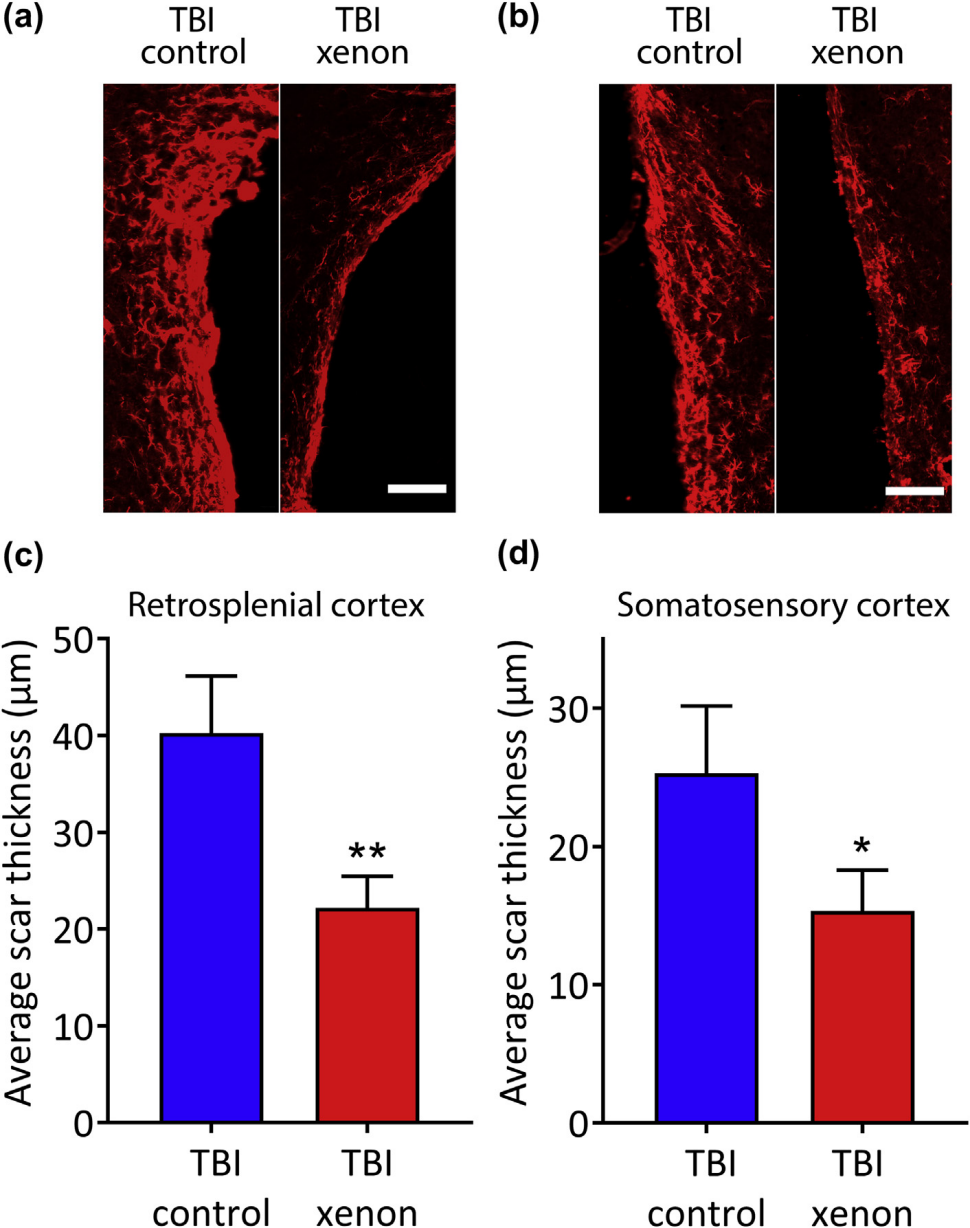
Xenon-treatment reduces astrocytic scarring 20 months after injury. Typical images of GFAP positive staining bordering lesion cavity in (a) retrosplenial cortex and (b) somatosensory cortex. Scale bars are 100 μm. The average thickness of the astrocytic scar bordering the lesion cavity was reduced in the TBI xenon group compared with the TBI control group in the (c) retrosplenial cortex and (d) somatosensory cortex. Control gas-treated animals (blue bars) received 75% nitrogen:25% oxygen. Xenon-treated animals (red bars) received 75% xenon:25% oxygen. Treatment was started 15 min after the impact and was administered for 3 h. The bars indicate mean values and the error bars are sem (*n*=7, TBI control; *n*=11, TBI xenon). *P<0.05; **P<0.01 compared with TBI control. GFAP, glial fibrillary associated protein; TBI, traumatic brain injury; sem, standard error of the mean.

We measured the thickness of the astrocytic scar bordering the lesion cavity in the retrosplenial cortex and somatosensory cortex (Fig. 4a and b) at 20 months after injury. The thickness of the astrocytic scar in the xenon-treated TBI group was significantly reduced compared with the TBI control group, by 45% (8%) (*P*<0.01) and 39% (8%) (*P*<0.05) in the retrosplenial cortex and somatosensory cortex, respectively (Fig. 4c and d).

### Xenon reduces the chronic increase in GFAP expression induced by TBI

**Fig. 5:**
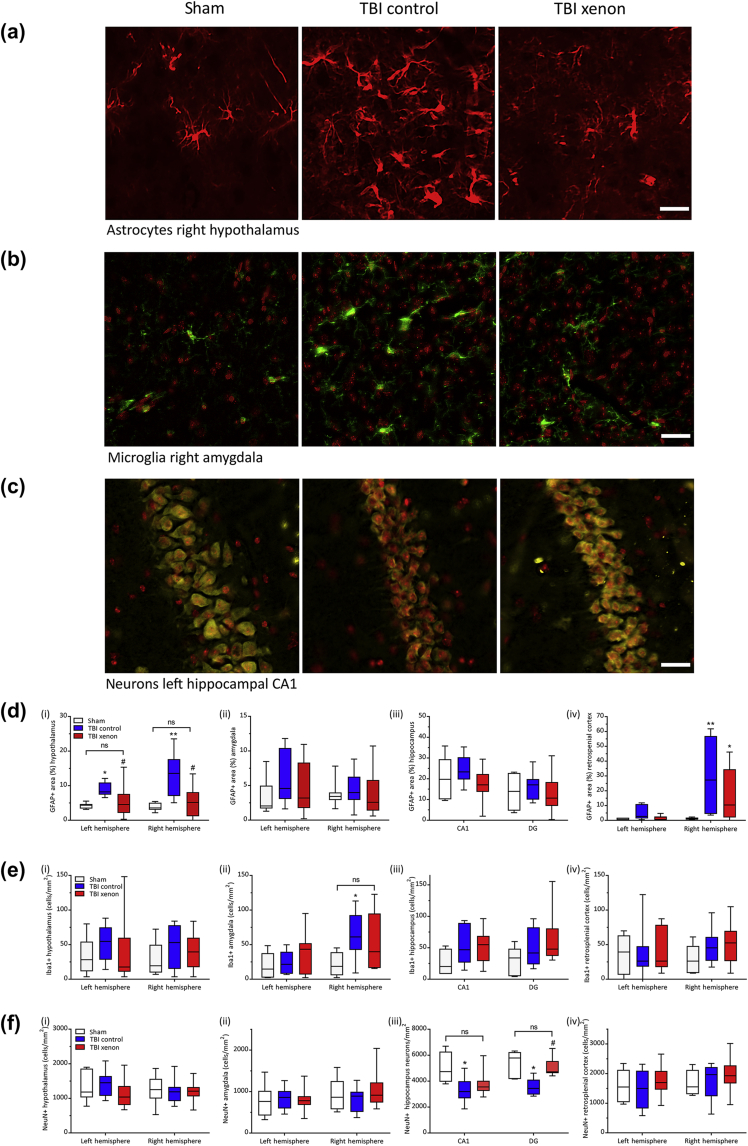
Xenon reduces neuroinflammation and neuronal loss 20 months after injury. Typical examples of immunostaining images for sham, TBI control and TBI xenon groups for (a) astrocytes (GFAP positive) in the hypothalamus, (b) microglia (Iba1 positive) in the amygdala, and (c) neurones (NeuN positive) in the CA1 region of the hippocampus. Scale bars in (a) and (c): 25 μm; scale Bar in (b): 30 μm. Quantification of: (d) GFAP positive area (*n*=7, sham; *n*=7, TBI control; *n*=11, TBI xenon, except for CA1: *n*=6, sham; CA1: *n*=6, TBI control); (e) number of Iba1 positive cells (*n*=6, sham; *n*=8, TBI control; *n*=11, TBI xenon, for all regions except the following hippocampal regions: CA1: *n*=5, sham; *n*=7, TBI control; *n*=10, TBI xenon; CA2: *n*=7, TBI control; DG: *n*=6, sham, *n*=8, TBI control, *n*=11, TBI xenon); and (f) number of NeuN positive cells in sham, TBI control and TBI xenon animals in (i) left and right hypothalamus, (ii) left and right amygdala, (iii) left hippocampus CA1 and DG, and (iv) left and right retrosplenial cortex (*n*=6, sham; *n*=8, TBI control; *n*=11, TBI xenon for all regions except the following: right retrosplenial cortex *n*=10, TBI xenon; right amygdala *n*=10, TBI xenon; CA1: *n*=4, sham, CA1: *n*=7, TBI control, CA1: *n*=10, TBI xenon, DG: *n*=5, sham, DG: *n*=6, TBI control, DG: *n*=9, TBI xenon). TBI control animals (blue boxes) and sham animals (white boxes) received 75% nitrogen:25% oxygen. TBI xenon animals (red boxes) received 75% xenon:25% oxygen. Treatment was started 15 min after the impact or sham procedure and was administered for 3 h. The boxes show median and inter-quartile range, the whiskers indicate the data range. *P<0.05, **P<0.01 compared with sham #*P*<0.05 compared with TBI control. GFAP, glial fibrillary associated protein; Iba1, ionised calcium-binding adapter molecule 1 ; NeuN, neuronal nuclei; TBI, traumatic brain injury; DG, dentate gyrus.

The GFAP stained area at 20 months after TBI was measured in four specific brain regions involved in contextual fear conditioning: the hypothalamus, amygdala, hippocampus and retrosplenial cortex.^33^, ^34^, ^35^, ^36^ We found significantly increased GFAP stained area in both ipsilateral and contralateral hypothalamus and in the right retrosplenial cortex in the TBI control group compared with the sham group (*P*<0.05 and *P*<0.01 for the different regions, respectively). In the TBI xenon group, GFAP positive area was significantly reduced (*P*<0.05) in the hypothalamus compared with the TBI control, with no significant difference between sham and TBI xenon groups (Fig. 5a and d). In the right retrosplenial cortex GFAP positive area was reduced in the xenon-treated TBI group compared with the TBI control group (Fig. 5d). We did not find significant differences between any of the groups in the other brain regions examined, amygdala or hippocampus (Fig. 5d; Supplementary Fig. S2a). In the sham group we observed differential expression of GFAP in different brain areas, with greatest expression in hippocampal CA1 and least in the retrosplenial cortex. Because we compared the same brain regions of the injured groups and sham group, variability between different brain regions did not impact the results.

### Xenon reduces microglial proliferation in the ipsilateral amygdala after TBI

We measured the number of Iba1-positive microglia in the hypothalamus, amygdala, hippocampus, and retrosplenial cortex at 20 months after injury (Fig. 5b and e). We observed a significant (*P*<0.05) increase in the number of Iba-positive microglial cells in the TBI control group in the right amygdala, whereas the TBI xenon group was not different to the sham group in this region (Fig. 5e). No other significant differences from sham were observed (Fig. 5e, Supplementary Fig. S2b).

### Xenon reduces neuronal loss in the contralateral CA1 and DG after TBI

We measured the number of NeuN-positive neurones in the hypothalamus, amygdala, hippocampus, and retrosplenial cortex (Fig. 5c and f). In the CA1 and DG of the hippocampus there was a significant (*P*<0.05) decrease in the number of neurones in the TBI control group compared with the sham group, whereas the TBI xenon group was not different to the sham group (Fig. 5f). In the hypothalamus, retrosplenial cortex, amygdala (Fig. 5f), and CA2 and CA3 regions of the hippocampus (Supplementary Fig. 2c), there was no significant difference in the number of neurones in the TBI groups or the sham group.

### Effect of xenon treatment on survival after TBI

**Fig. 6:**
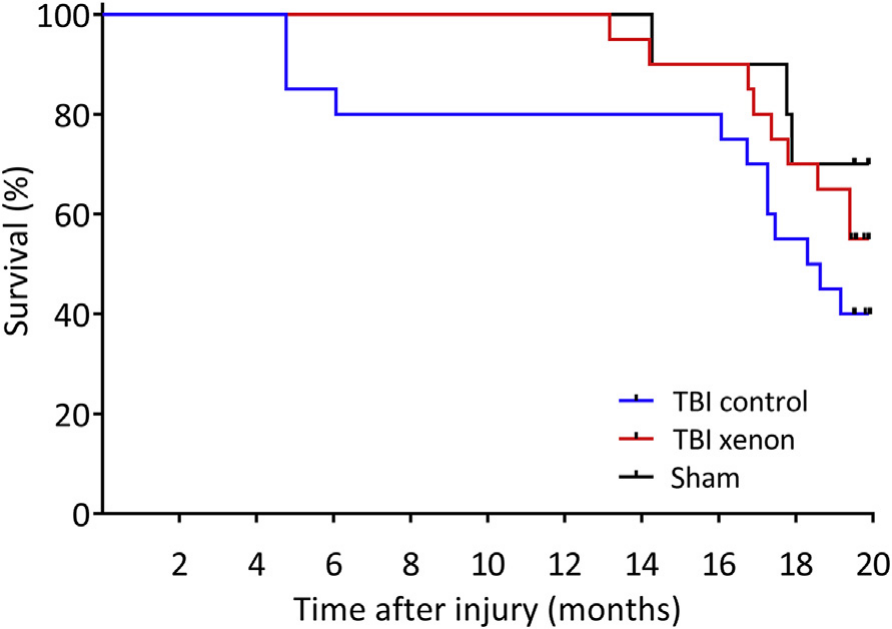
Kaplan–Meier survival curves up to 20 months after injury. The untreated TBI control animals started to die earlier, with 20% mortality at 12 months, whereas in the TBI xenon group and sham groups there was 100% survival up to 12 months. At 12 months after injury the survival curve for the TBI xenon group was significantly different (*P*<0.05) to the TBI control group. At the end of the observation period mortality was 60% in TBI control, 30% in sham and 45% in TBI xenon groups. At 20 months the survival curve for the xenon-treated group was not significantly different to the TBI control group (*P*=0.09). TBI control (blue line) and sham operated (black line) animals received 75% nitrogen:25% oxygen; TBI xenon (red line) received 75% xenon:25% oxygen. Treatment was started 15 min after the impact or sham procedure and was administered for 3 h. Beginning of observation period: *n*=10, sham; *n*=20, TBI control; *n*=20, TBI xenon; End of observation period: *n=*7, sham; *n*=8, TBI control; *n*=11, TBI xenon. TBI, traumatic brain injury.

Our hypotheses were that xenon treatment improves survival after TBI at 12 and 20 months after injury. All of the deaths that occurred up until the end of the observation period of 20 months were spontaneous. We analysed the data using Kaplan–Meier survival curves (Fig. 6). At both the 6 and 12 month time points, there was 20% mortality in the TBI control group whereas in the sham and TBI xenon groups mortality was 0%. The survival curve for the TBI xenon group was significantly different (*P*<0.05) to that of the TBI control group at 12 months. Animals in the TBI control group were more likely to die than the TBI xenon group in the first 12 months with a hazard ratio of 8.38 (95% confidence interval [CI], 1.13–61.95). At the end of the observation period of 20 months, the mortality in the TBI control group was 60%, whereas in the sham group it was 30% and in the TBI xenon group it was 45%. At the end of the observation period (20 months), the survival curves for the TBI control and TBI xenon groups were not significantly different (*P*=0.09); and the hazard ratio was 1.68 (95% CI, 0.70–4.00). The data as a whole show that xenon treatment significantly improves survival in the first 12 months after TBI.

## Discussion

TBI is recognised as a dynamic condition with long-lasting sequelae that evolve in the weeks, months, and years after injury.^1^ Many of the long-term cognitive and motor impairments experienced by TBI patients result from the developing secondary injury arising after the primary insult. Survivors of TBI have a significantly increased risk of death,^4^ and it is known that even a single TBI early in life is a risk factor for developing AD, chronic traumatic encephalopathy (CTE), and other cognitive impairments later in life.^6^ The mechanistic link between TBI, AD, and CTE is not fully understood, but chronic neuroinflammation and neurodegeneration are thought to play key roles.^6^ Our aim was to investigate the effects of xenon treatment on secondary injury in both the short- and very long-term on behavioural outcomes longitudinally in an ageing cohort, and on survival, after a single TBI.

### Experimental paradigm

The rodent CCI model is a widely used, highly reproducible, pre-clinical model of blunt TBI.^37^ With a few notable exceptions,^29^, ^38^, ^39^, ^40^ the majority of studies using this model have investigated outcomes in the time frame of 1–28 days. Our current study is one of the longest to evaluate a neuroprotective treatment in an animal model of TBI as the study duration was very close to the end of a healthy animal's life span. The CCI injury parameters we used result in a clear early secondary injury, evidenced by the lesion volume approximately doubling in the first 24 h. This early secondary injury is accompanied by a moderate neurological impairment at this time point.

### Xenon treatment after TBI reduces acute secondary injury and improves short-term neurological impairment

Our results show that xenon is effective in significantly reducing the developing secondary injury in the first 24 h. We also found a significant improvement in acute neurological outcome at 24 h in the xenon-treated group. These results are consistent with a previous study where we showed that xenon reduced total contusion volume, but we did not examine secondary injury separately.^16^

### Xenon treatment prevents late-onset memory deficits after TBI

A single TBI early in life increases the risk of cognitive decline in later life.^6^, ^41^ We examined a cognitive function, hippocampus-dependent memory, in the same cohort, at 2 weeks and 20 months after traumatic injury. At the 2 week time point, no memory impairment was evident in either the TBI control or TBI xenon groups. In contrast, at 20 months in the TBI control group there was a significant memory impairment compared with the uninjured sham group. Remarkably, this late-onset cognitive impairment was absent in the TBI xenon group. The contextual fear conditioning paradigm we used is robust and one of the most comprehensively characterised rodent models of learning and memory. This test allowed us to use novel visuospatial contexts for the two time points and is not confounded by motor deficits. Although memory is a cognitive function, it is only one component of global cognition that can be assessed in patients. Nevertheless, our findings are of particular clinical relevance because of the association of TBI with increased risk of AD and other neurodegenerative conditions in which delayed onset memory impairment is observed.^6^, ^42^ Our results suggest that xenon treatment may prevent development of these persistent cognitive deficits in TBI patients.

### Histopathological correlates of late-onset cognitive impairment are reduced by xenon

Long-term loss of cerebral white matter after TBI is associated with cognitive impairment in patients. In the TBI control group we observed chronic neurodegeneration of white matter in the contralateral corpus callosum that was associated with reactive astrogliosis and an increase in microglia, similar to findings reported by Pischiutta *et al*^29^ and Loane *et al*^39^ 12 months after CCI in mice. Remarkably xenon treatment reduced loss of white matter and chronic astrogliosis in the corpus callosum. Interestingly, microglia were significantly increased in the corpus callosum of both TBI control and TBI xenon groups, suggesting involvement of toxic A1-type reactive astrocytes^43^ induced by activated microglia in the chronic neurodegeneration. In addition to reducing reactive astrogliosis in the contralateral corpus callosum, xenon treatment resulted in a significant reduction in the thickness of astrocytic scarring bordering the lesion cavity in the retrosplenial and somatosensory cortex. We suggest that the reduced scarring limits neurodegeneration and promotes axonal repair in the perilesional tissue.

The neuronal circuit involved in contextual fear conditioning involves a number of brain regions, including the hippocampus, amygdala, retrosplenial cortex, and hypothalamus.^33^, ^34^, ^35^, ^36^ We found neuronal loss in the CA1 and DG regions of the contralateral hippocampus in the TBI control group 20 months after TBI, consistent with the results of another study at 12 months after injury.^39^ Both of these regions are involved in episodic memory, and this neuronal loss in the TBI control group correlates with the late-onset memory impairment. Remarkably, xenon treatment reduced this long-term neurodegeneration, with the number of neurones in the CA1 region and the DG regions in the xenon-treated TBI group not different to the sham group, correlating with xenon preventing late-onset impairment of hippocampus-dependant memory. We found increased astrocyte reactivity in the hypothalamus and retrosplenial cortex of TBI control animals compared with the sham group. This is consistent with similar findings reported in the thalamus and cortex of mice 1 yr after CCI injury.^29^ Interestingly, in xenon-treated animals, the GFAP positive area was decreased in the retrosplenial cortex and was not different from the sham group in the hypothalamus. Hypothalamic dysfunction has been found in other rodent models of TBI,^44^, ^45^ including evidence of persistent hypothalamic astrocytosis 2 months after CCI.^46^ However, we did not observe significant changes in astrocyte reactivity in the amygdala or hippocampus. We observed increases in number of microglia in the right amygdala in the TBI control group but not in the xenon-treated group. The amygdala is involved in memory recall involving anxiety and fear responses, and this observation may play a role in xenon preventing long-term cognitive impairment. Our findings suggest that the learning and memory deficits developing late after the injury result from a combination of discrete injury, involving key brain areas such as CA1 and DG regions of the hippocampus, the hypothalamus, retrosplenial cortex, and amygdala, and reduction of connectivity resulting from loss of white matter. Overall, xenon treatment preserved neuronal white matter, and reduced neuronal loss in the hippocampal CA1 and DG regions, and this may explain how xenon treatment prevents the late-onset cognitive impairment. Xenon reduced chronic GFAP immunoreactivity, a well-known marker of astrocyte activation and neuroinflammation^31^ in the hypothalamus and retrosplenial cortex. Given the recent evidence of the role astrocytes play in regulating neuronal activity, it is worth noting that even in the absence of cytotoxic effects, changes in astrocyte number and reactivity may result in neuronal dysfunction. Hypothalamic dysfunction associated with TBI is not yet fully understood; however, this is known to contribute to cognitive deficits in TBI patients,^47^, ^48^ and disruption of sleep and circadian rhythm are observed in many TBI patients.

### Xenon treatment improves survival after injury

TBI is associated with an increased risk of death in those who survive the initial injury. A longitudinal study following a large cohort of patients for up to 13 yr after TBI found that by the end of the study mortality was 40%.^3^, ^4^ When compared with a control group, this represents an increase in mortality of 2.8-fold overall (all ages) and a 7-fold increase for those sustaining a TBI at 15–54 yr.^34^ We aimed to determine whether xenon treatment would increase survival in a cohort of mice that had sustained a single TBI early in life. Our cohort was aged 2.5 months (young adult) when they were subjected to CCI, and our objective was to study them until old age. Given the reported life span of male C57BL/6 mice of 23–30 months,^49^, ^50^ we studied them for 20 months after injury (age 23 months), and determined whether there was an effect of treatment at 12 months and at the end of the study. We observed a greater number of deaths in the TBI control group compared with the TBI xenon group or sham group at all time points. Early deaths (6 months after injury) were observed in the TBI control group but not in any other group, which we attribute to an increased risk of death resulting from the TBI. Xenon treatment improved survival significantly in the first 12 months after injury. Interestingly, no animals in the TBI xenon group or the sham group died in the first 12 months. At 12 months the hazard ratio of the TBI control group compared with the TBI xenon group was 8.34, similar to the 7-fold increased risk of death in TBI patients suffering a TBI early in life. At 20 months there were more survivors in the xenon-treated group, with a hazard ratio of 1.68 (*P*=0.09). The lack of a significant difference between TBI control and TBI xenon groups at the end of the study likely reflects that this time point is very close to the normal expected life span. Nevertheless, these results show that xenon improves early survival after TBI, and further preclinical studies to investigate this are merited.

### Clinical relevance

Our aim was to evaluate xenon's potential as a treatment for TBI in clinically relevant settings, with xenon treatment 15 min after the trauma, based on a scenario where a TBI patient might receive medical attention at the scene of injury within 15–20 min. Although there may be limited circumstances when xenon could be administered within this time frame, it is possible that xenon could be given by first responders. The most likely treatment scenario would be on arrival at hospital, and xenon is effective even when treatment start time is delayed up to 3 h after injury.^16^ The 3 h duration of xenon treatment used is relatively short, and it is plausible that extending treatment time might result in greater neuroprotection. The mechanisms by which xenon reduces secondary injury may be pleotropic, but preventing or reducing glutamate excitotoxicty^51^, ^52^, ^53^, ^54^, ^55^, ^56^ is likely to play an important role. Glutamate levels in TBI patients peak 24–72 h after injury.^57^ It is possible that xenon treatment given during the entire period that glutamate is elevated would be more efficacious. Current treatment for TBI patients is largely supportive, with no clinically proven treatments specifically targeting neuronal loss and neurodegeneration.^11^ Xenon is approved for clinical use as a general anaesthetic and has recently undergone clinical trials as a neuroprotectant for ischaemic brain injury in neonates and adult cardiac arrest patients.^12^, ^58^, ^59^ We show here that xenon treatment after TBI in mice reduces chronic neurodegeneration of cerebral white matter, reduces loss of hippocampal neurones, prevents late-onset TBI-related cognitive impairment, and improves long-term survival. These long-term improvements in clinically relevant outcomes and survival provide further support to the idea that xenon could be used as an early neuroprotective treatment in TBI patients.

## Authors' contributions

Study design/planning: RCP, KR, SCT, RD.

Study conduct: RCP, TH, KR, RD.

Data analysis: all authors.

Drafting of the paper: RCP, SCT, RD.

Revision of the paper: all authors.

## Figures and Tables

**figs1:**
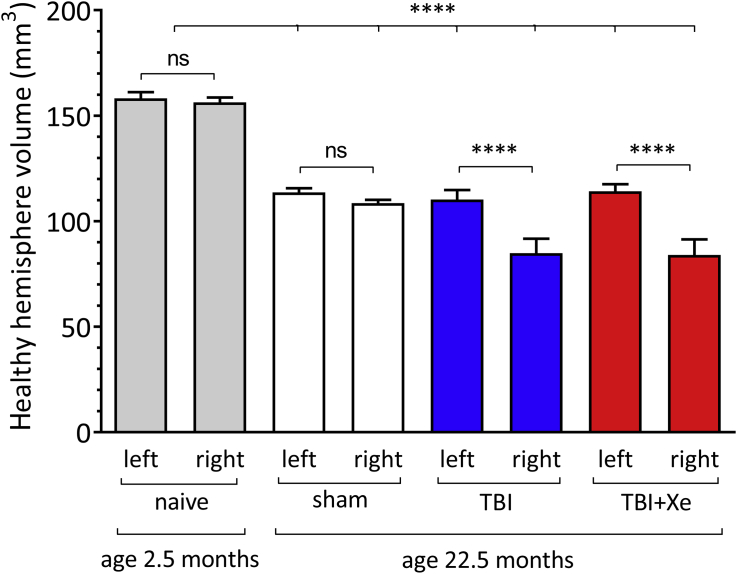
Healthy brain hemisphere volumes in naïve animals aged 2.5 months (grey bars) and in sham (white bars), TBI control (blue bars) and TBI xenon groups (red bars) aged 22.5 months. TBI xenon animals received 75% xenon:25% oxygen. Sham and TBI control animals received 75% nitrogen:25% oxygen. Treatment was started 15 minutes after the impact or sham procedure and was administered for 3 hours. Bars represent mean values and error bars are standard errors (*n*=5, naïve; *n*=7, sham; *n*=8, TBI control; *n*=11, TBI xenon). ∗∗∗∗*P*<0.0001.

**figs2:**
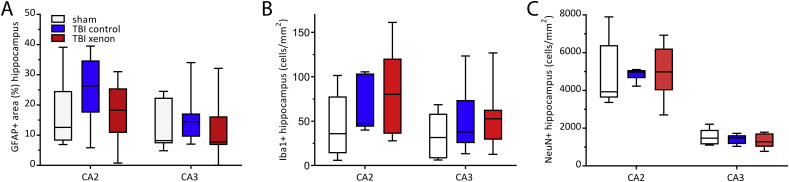
Quantification of reactive astrocytes (GFAP positive area) **(A)**, number of microglia (Iba1 positive) **(B)** and number of neurons (NeuN positive), **(C)** in the hippocampal CA2 and CA3 areas, 20 months after injury. TBI control animals (blue bars) and sham animals (white bars) received 75% nitrogen:25% oxygen TBI xenon animals (red bars) received 75% xenon:25% oxygen. Treatment was started 15 minutes after the impact or sham procedure and was administered for 3 hours. The boxes show median and interquartile range, the whiskers indicate the data range (GFAP: *n*=7, sham; *n*=7, TBI control; *n*=11, TBI xenon; except CA2: *n*=10, TBI xenon. Iba1: *n*=6, sham; *n*=8, TBI control; *n*=11, TBI xenon, for all regions except CA2: *n*=7, TBI control; NeuN: *n*=6, sham; *n*=8, TBI control; *n*=11, TBI xenon, for all regions except CA2: *n*=5, sham; CA2: *n*=6, TBI control; CA2: *n*=10, TBI xenon).
